# Whitlow

**Published:** 1910-04-16

**Authors:** F. C. Pybus

**Affiliations:** late Surgeon to out-Patients' Dressing Department, Royal Victoria Infirmary, Newcastle-on-Tyne.


### The General Practitioner's Column.

[Contributions to this Column are invited, and, if accepted, will be paid for.]

### WHITLOW.

By F. C. PYBUS, M.B., B.S., F.R.C.S.; late Surgeon to out-patients' Dressing Department, Royal Victoria Infirmary, Newcastle-on-Tyne.

### I.—VARIETIES AND DIAGNOSIS.

In considering the varieties of whitlow, one must note the various tissues of the finger in order of depth, where inflammation often progressing to suppuration may occur. Taking the term cuticle to comprise the whole of the epidermal portion of the skin, we meet with the five following varieties.

*Cuticular.*—Corresponding in situation to a blister, raised by the effect of pressure or heat. This variety is usually painless, spreads rapidly round, and in some cases over the whole area of the finger. In many cases it is multiple, affecting several fingers, and prone to occur in people liable to prick themselves, and in such conditions as syringomyelia and Raynaud's disease. The base of the abscess is covered by epithelium (the separation taking place about the stratum granulosum) so that after incision and removal of the separated tissue no scar results. Should the pus remain long in contact with the deep epithelium this becomes destroyed, and the second variety, or *subcuticular* follows, almost corresponding to the third degree of a burn where the whole epithelium is destroyed, and the tops of the dermal papillæ exposed. This form becomes painful and must heal by granulation.

*Subcutaneous.*—This form commences as a cellulitis and may remain localised, or spread further in the subcutaneous tissue of the finger and palm of the hand. Should the infection extend deeply, the structure involved depends on the site of the primary focus. Over the terminal phalanx the periosteum is the nearest structure, and when this is infected it becomes stripped from the bone resulting in the *subperiosteal* abscess, and leading, as a rule, to an acute necrosis of the terminal phalanx.

Over the remaining phalanges the tendon sheath becomes invaded leading to a thecal inflammation and suppuration, so that *thecal* whitlow represents a suppurative teno-synovitis. From any of the latter three forms the remaining phalanges or joints may become infected.

Although the above varieties may occur separately, in a larger proportion of cases, a combination of two or more forms obtains, so that the finger in the worst cases becomes totally disorganised.

The following combinations are the more common:—

1. A subcutaneous abscess over the terminal phalanx resulting in the subperiosteal form, and leading to necrosis of the terminal phalanx, and often disorganisation of the terminal joint.
2. A subcutaneous abscess of small size with extensive separation of the epidermis.
3. A subcutaneous abscess with infection of the tendon sheath—this being the more common origin of thecal whitlow than a direct wound and infection. A careful examination of a number of fingers showed that infection may spread round the sheath on its outer aspect and so infect the adjacent bones and joints. In rare cases the subperiosteal whitlow may arise as an acute epiphysitis, this of necessity being limited to children and young adults.

*Symptoms.*—A history can usually be obtained of some wound, often of small size—as a pin prick; in other cases a suppurating wound is readily found. The classical signs of inflammation are present in all the deeper forms. The cuticular variety rarely shows any marked evidence of redness, tenderness, or pain. The finger is, as a rule, swollen, the situation of this depending on the variety present; œdema is usually found on the dorsum of the finger and hand owing to the lax tissues in this situation.

Redness may be diffuse or localised. Pain of an intense aching character, often throbbing, is more marked when the hand is allowed to hang down, and may be sufficient to prevent sleep. Tenderness and inability to use the finger are also present.

*Diagnosis.*—It is of great importance to distinguish between the different varieties, as their treatment varies considerably in detail. The cuticular variety usually presents itself as a yellow blister, soft and fluctuating, situated on one side of the finger, or may entirely surround it. The yellow area may be surrounded by a hyperæmic zone. There is rarely pain. In some cases the nail may become separated, and by cutting round the base of the separated cuticle the whole covering of the finger may be removed.

The subcuticular may be associated with the above; more often it follows a wound involving the subcutaneous tissue, so that one has the signs of a subcutaneous abscess with separation of the surrounding epidermis. The condition gives rise to some pain, swelling, and tenderness.

The signs of a subcutaneous whitlow are those of a cellulitis, usually starting on the palmar surface of the finger, and may be limited to the terminal phalanx, or spread around the whole finger and into the palm. The finger becomes red, swollen, and tender, œdema is present on the dorsum of the finger, and in the later stages softening and fluctuation occur. The pain is not so acute as in the next two varieties, and the finger still possesses some power of movement.

The finger in the commonest or subperiosteal variety is markedly swollen at its extremity, red, tender, and there may be a sense of fluctuation over the palmar surface. When opened or after spontaneous bursting a sinus remains, at the bottom of which the terminal phalanx can be felt, bare or covered by slough. The edges of the wound become everted and filled with protuberant granulations. If necrosis has occurred, in time the phalanx separates, usually coming away with a small slough of tendon which is inserted into it. If the subcutaneous abscess is opened early before the whole periosteum is separated, the bone may escape destruction or only the terminal portion may die.

In the thecal variety the finger is swollen, extremely painful, tender, and markedly rigid. The slightest movement causes great pain. The swelling hardly involves the terminal phalanx, the remainder being swollen, œdema is present over the dorsum of the finger and hand. In the case of the index, middle, and ring fingers, the swelling is sharply limited at the level of the heads of the metacarpal bones. In the little finger and thumb the swelling may extend; in the latter case along the thenar eminence towards the wrist, in the former along the ulnar border of the hand into the palm where the common synovial sheath may be involved. Inability to flex the terminal phalanx is sometimes a useful sign.

In the worst cases the finger may be much enlarged, hard, and immobile, while sinuses exist at several places, especially over the course of the tendons on the palmar side and over the joints on the dorsal aspect. This means that the tendons have probably sloughed, and that septic arthritis is present.

*(To be continued).*

